# Prevalence and Genetic Characterization of Methicillin-Resistant *Staphylococcus aureus* Isolated from Pigs in Japan

**DOI:** 10.3390/antibiotics13020155

**Published:** 2024-02-04

**Authors:** Michiko Kawanishi, Mari Matsuda, Hitoshi Abo, Manao Ozawa, Yuta Hosoi, Yukari Hiraoka, Saki Harada, Mio Kumakawa, Hideto Sekiguchi

**Affiliations:** Veterinary AMR Center, National Veterinary Assay Laboratory, Ministry of Agriculture, Forestry and Fisheries, Tokyo 185-8511, Japan; mari_matsuda510@maff.go.jp (M.M.); manao_ozawa500@maff.go.jp (M.O.); yukari_hiraoka970@maff.go.jp (Y.H.); saki_harada360@maff.go.jp (S.H.); mio_kumakawa230@maff.go.jp (M.K.);

**Keywords:** LA-MRSA, ST398, ST8, antimicrobial resistance, zinc resistance, SNP analysis

## Abstract

We investigated the prevalence of livestock-associated methicillin-resistant *Staphylococcus aureus* (LA-MRSA) in pig slaughterhouses from 2018 to 2022 in Japan and the isolates were examined for antimicrobial susceptibility and genetic characteristics by whole-genome analysis. Although the positive LA-MRSA rates on farms (29.6%) and samples (9.9%) in 2022 in Japan remained lower than those observed in European countries exhibiting extremely high rates of confirmed human LA-MRSA infections, these rates showed a gradually increasing trend over five years. The ST398/t034 strain was predominant, followed by ST5/t002, and differences were identified between ST398 and ST5 in terms of antimicrobial susceptibility and the resistance genes carried. Notably, LA-MRSA possessed resistance genes toward many antimicrobial classes, with 91.4% of the ST398 strains harboring zinc resistance genes. These findings indicate that the co-selection pressure associated with multidrug and zinc resistance may have contributed markedly to LA-MRSA persistence. SNP analysis revealed that ST398 and ST5 of swine origin were classified into a different cluster of MRSA from humans, showing the same ST in Japan and lacking the immune evasion genes (*scn*, *sak*, or *chp*). Although swine-origin LA-MRSA is currently unlikely to spread to humans and become a problem in current clinical practice, preventing its dissemination requires using antimicrobials prudently, limiting zinc utilization to the minimum required nutrient, and practicing fundamental hygiene measures.

## 1. Introduction

*Staphylococcus aureus* is a Gram-positive catalase-positive bacterium that is commonly found on the skin and mucosa of humans and animals [1,2]. Although usually classified as a commensal bacterium, *S. aureus* is a facultative pathogen that can cause several diseases, ranging from mild skin lesions to severe and potentially fatal infections [3].

Penicillin G (natural penicillin), a successfully mass-produced antibacterial drug in the 1940s, was effective against *S. aureus*, but its antibacterial potential became ineffective due to *S. aureus*’ production of penicillinase. Therefore, methicillin was developed and has been used in Europe and the United States since around 1960; methicillin-resistant *S. aureus* (MRSA) was isolated shortly after methicillin introduction [4]. The WHO has now designated MRSA as a high-priority pathogen given the emergence of its strains [5].

MRSA is the leading cause of hospital- and community-associated infections worldwide, with livestock-associated MRSA (LA-MRSA) emerging across Europe and many other parts of the world [6,7,8,9,10]. MRSA isolates from clonal complex 398 (CC398) have increasingly proliferated at high rates among pigs reared in European countries [11,12,13]. 

Different regions and countries exhibit varying positivity rates of LA-MRSA detected in pig farms, with European countries, specifically Denmark and the Netherlands, having extremely high positive rates of MRSA (over 75% or more) on pig farms [14,15].

A comparison of the molecular typing results of LA-MRSA isolates in pigs showed regional differences in genetic variant distribution. In Europe, the United States, and Canada, the primary LA-MRSA strain found in pig production farms was CC398. The most common *spa* types found in Europe in the CC398 lineage were t011, t034, and t108, which were mainly identified in pig breeding and production farms [6].

In Asia, non-CC398 LA-MRSA strains have been reported in pigs, whereas LA-MRSA CC9 strains have mostly been identified in pig farms in China, Thailand, Pakistan, and Vietnam [6,10].

In Japan, MRSA ST221, ST97, and ST5 were isolated from nasal samples from pig slaughterhouses in eastern Japan between 2009 and 2013 [16,17]. Thereafter, MRSA ST398 was isolated from pig farms in the eastern part in 2012 and from pig slaughterhouses in the northern part in 2017 [18]. In an epidemiological study, MRSA ST398/t034 was isolated from all sources in slaughterhouses, diseased pigs on farms, imported breeding pigs, and farm dust [19]. LA-MRSA isolates are human-transmittable, with people working or living on pig farms, including farmers and their families, abattoir staff, and veterinarians, being at a higher risk of contracting LA-MRSA from pigs [20,21]. In a 2013 survey of European Union/European Economic Area countries, LA-MRSA accounted for 3.9% of human MRSA isolates, whereas in five countries (Belgium, Denmark, Spain, the Netherlands, and Slovenia), it was over 10% [22].

LA-MRSA harbors genes encoding *mecA* that impart resistance to methicillin and other β-lactam antibiotics, such as cephalosporins, penicillins, and carbapenems. In addition, a wide range of resistance genes were detected in LA-MRSA, and these include genes known to be common in other *Staphylococcus* types of human and animal origin, such as the tetracycline resistance genes *tet*(M), *tet*(K), and *tet*(L), the macrolide resistance genes *erm*(A), *erm*(B), and *erm*(C), the trimethoprim resistance genes *dfrK* and *dfrG*, and the chloramphenicol resistance genes *fexA* and *cfr*. Heterogeneity in LA-MRSA resistance profiles has also been demonstrated [23,24,25].

Reports on human-isolated CC398 MRSAs in Japan are few [26,27], and studies reporting human-isolated LA-MRSA types remain nonexistent. However, as countries with high positive rates in pig farms have high LA-MRSA infection rates in humans, monitoring LA-MRSA prevalence in pig farms is crucial. Therefore, we investigated MRSA-positive rates in pig farms in Japanese slaughterhouses. We also examined antimicrobial susceptibility and genetic profiles such as ST, *spa* types, SCC*mec* types, antimicrobial resistance genes, zinc resistance genes, and virulence genes. This was carried out to identify LA-MRSA characteristics in Japan and to formulate countermeasures to prevent LA-MRSA transmission from pigs to humans and from humans, pigs, and other sources to pigs.

## 2. Results

### 2.1. Prevalence of MRSA in Slaughterhouses

The MRSA positivity rate of the samples for all slaughterhouses surveyed was 2.9% in 2018, increased significantly to 6.4% in 2019, and continued to increase thereafter, reaching 9.9% in 2022. The MRSA positivity rate of farms for all slaughterhouses surveyed was 8.3% in 2018, increased significantly to 20.4% in 2021, and continued to increase thereafter, reaching 29.6% in 2022 (Table 1). 

The rate of positive MRSA samples in the two slaughterhouses studied continuously for over 5 years was 2.9% in 2018, increased significantly to 6.7% in 2019, and continued to increase thereafter to 9.9% in 2022. The MRSA positivity rate of farms in the same two slaughterhouses was 8.3% in 2018 and continued to increase thereafter to 23.4% in 2022 (Table 2).

### 2.2. Molecular Characterization of MRSA Isolates 

Here, 88 MRSA isolates underwent whole-genome analysis for characterization (see Table 3 and Table S1). ST398 (66.0%) was the predominant MLST type, followed by ST5 (27.3%). Other types identified were ST8, ST380, ST7096, and ST8632. The *spa* type was *t*034 (54.5%) in ST398 and *t*002 (11.4%) and *t*21388 in ST5 (11.4%). SCC*mec* type Vc (5C2&5) (45.45) was predominant in ST398, whereas type IV (2B) (13.6%) was predominant in ST5.

### 2.3. Antimicrobial Susceptibility and the AMR Gene

Resistance rates were observed for tetracyclines: tetracycline (TC) (85.2%), macrolides: azithromycin (AZM) (46.6%) and erythromycin (EM) (46.6%), phenicol: chloramphenicol (CP) (43.2%), and aminoglycosides: streptomycin (SM) (31.8%). Resistance to fluoroquinolone: ciprofloxacin (CPFX) (18.2%) was also observed. ST398 presented markedly higher TC resistance rates than ST5, but ST5 exhibited markedly higher AZM, EM, CP, and CPFX resistance rates than ST398 (Table 4).

ST398 and ST5 exhibited differences in AMR gene rates and resistance gene types (Table 5).

For ST398, 58 isolates (100%) possessed one or two tetracycline-resistant genes (*tet*(K), *tet*(L), *tet*(M)), whereas only 14 isolates (58.3%) of ST5 possessed one of the tetracycline-resistant genes (*tet*(K), *tet*(L)) and none of the ST5 isolates had *tet*(M). 

Regarding macrolide resistance genes, 25 (43.1%) of the ST398 isolates possessed one resistance gene (*erm*(A) or *erm*(C)) compared with 17 (70.8%) of the ST5 isolates that possessed one gene (*erm*(C)). None of the ST5 isolates possessed *erm*(A). 

For lincosamide/streptomycin resistance genes, 62.1% of the ST398 isolates possessed *ls*a(E) + *lnu*(B), whereas ST5 harbored 100% of the *vag*(A) gene and two isolates (8.3%) also carried the *lasa*(E) + *vag*(A) gene.

ST398 and ST5 harbored the phenicol-resistant gene *fex*A in 20 (34.5%) and 12 (50%) isolates, respectively. Only the ST398 isolate possessed the *catA* gene; the ST5 isolate did not.

Furthermore, 45 (77.6%) of the ST398 isolates had aminoglycoside resistance genes (*ant(9)-Ia*), but ST5 isolates had no *ant(9)-Ia*; 8 (33.3%) of the ST5 isolates had *aadD1*. 

ST398 and ST5 harbored the trimethoprim-resistant gene, *dfrG*, in 46 (79.3%) and 3 (12.5%) isolates, respectively.

Four mutations, GyrA_S84A, GyrA_S84L, ParC_S80F, and ParC_S80Y, to quinolone resistance were identified: 13 (54.2%) ST5 isolates harbored at least one mutation, with GyrA_S84A + ParC_S80F (7/29.2%) being the major mutation.

### 2.4. Zinc Chloride Resistance, PVL Toxin, and Bacteriophage-Mediated Immune Evasion Genes

Among the ST398 isolates, 53 (91.4%) possessed the zinc resistance gene, whereas only 2 (8.3%) of the ST5 isolates harbored this gene. All isolates lacked PVL toxin genes (lukF-PV and lukS-PV), whereas all ST5, ST8, ST380, and ST7096 isolates harbored leukotoxin genes (lukD and lukE). 

ST398 and ST5 isolates had no *scn*, *sak*, or *chp* genes. However, the ST8 isolate possessed the *scn* and *sak* genes, and ST7096 possessed these three genes.

### 2.5. Phylogenetic Analysis

**Figure 1:**
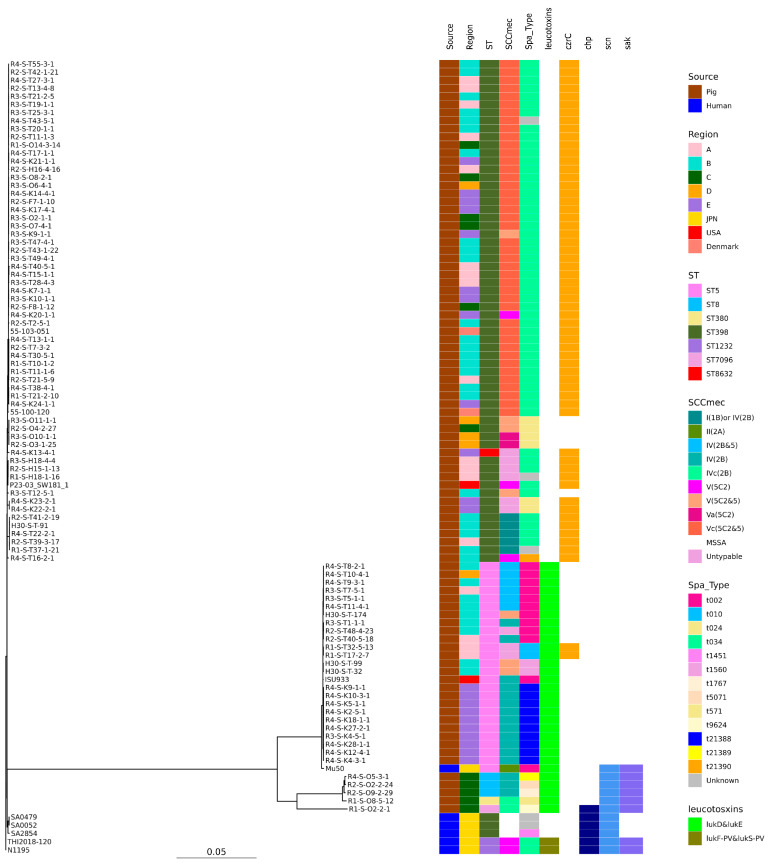
Phylogenetic tree of the 88 isolates from this study and 10 genomes available in GenBank. N1195 isolated from a human patient in Japan was used as the reference for the construction of the tree. Isolation source, country or region, ST, SCC*mec*, *Spa* type, leukotoxin genes (lukD and lukE, lukF-PV and lukS-PV), and immune evasion genes (*scn*, *sak*, *chp*) are also shown with color coding.

SNP-based phylogenetic tree analysis revealed that these isolates were clearly clustered by ST (Figure 1).

**Figure 2:**
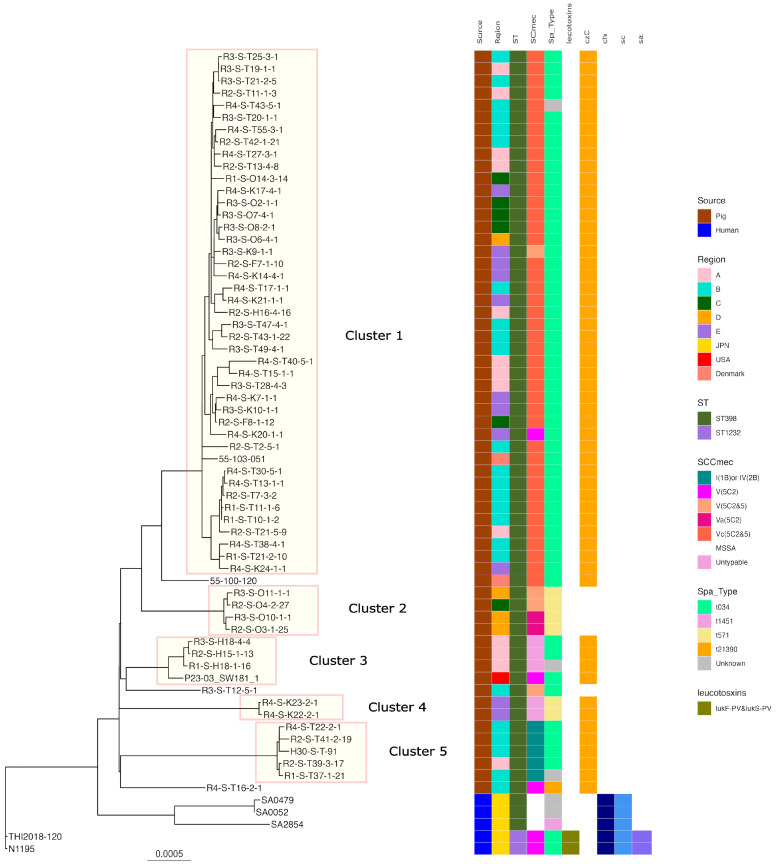
Phylogenetic tree of the ST398 isolates from this study and genomes available in GenBank. N1195 isolated from a human patient in Japan was used as the reference for the construction of the tree. Isolation source, country or region, ST, SCC*mec*, *Spa* type, leukotoxin genes (lukD and lukE, lukF-PV and lukS-PV), and immune evasion genes (*scn*, *sak*, *chp*) are also shown with color coding.

Regarding ST398, five clusters were formed when SNP analysis clustered strains with fewer than 100 base differences. Cluster 1 had the highest number of strains with t034-SCC*mec*V and Vc belonging to it, including isolates from all regions of Japan and Europe. While isolates of *t*571*t* without zinc resistance genes (Cluster 2) were isolated from the C and D regions *t*034-*t*034-SCC*mec*-untypable, isolates of t034- from the A region and *t*0571 from the USA belonged to Cluster 3. Isolates belonging to Clusters 4 and 5 were also isolated from a limited number of regions. All human-isolated strains were identified in different clusters to which the pig-derived strains belonged (Figure 2).

**Figure 3:**
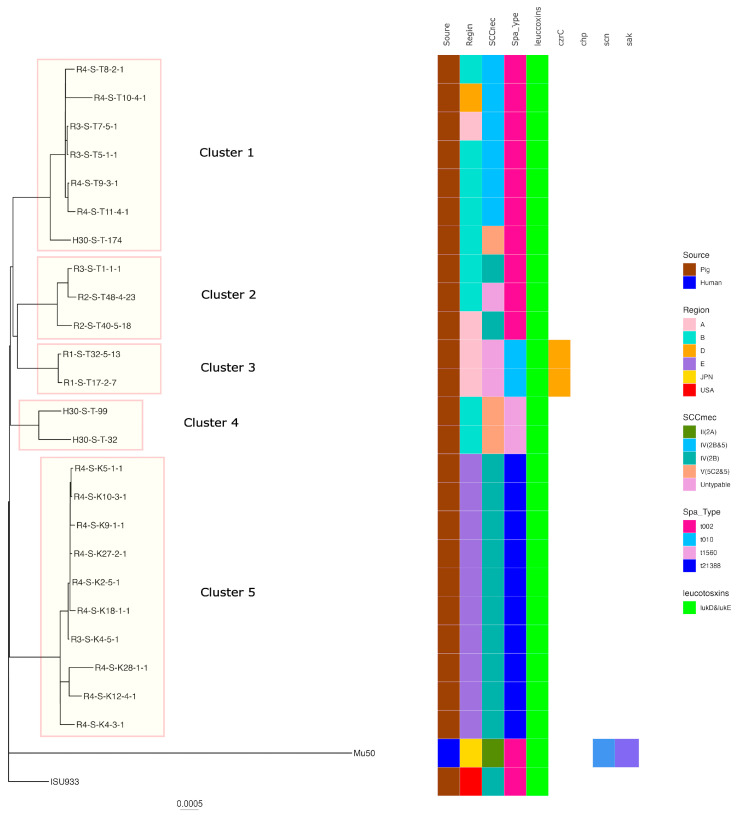
Phylogenetic tree of the ST5 isolates from this study and genomes available in GenBank. Mu50 isolated from a human patient in Japan was used as the reference for the construction of the tree. Isolation source, country or region, ST, SCC*mec*, *Spa* type, leukotoxin genes (lukD and lukE, lukF-PV and lukS-PV), and immune evasion genes (*scn*, *sak*, *chp*) are also shown with color coding.

Regarding ST5, five clusters were formed when the SNP analysis clustered strains with fewer than 100 base differences. Cluster 5 had the highest number of strains of *t*21388-SCC*mec*IV(2B) belonging to it, including isolates from regions of E. The isolates belonging to the other clusters were also isolated from a limited number of regions. Pig-derived strains isolated in the USA were identified in different clusters to which the Japanese pig-derived isolates belonged. In addition, human-isolated strains were identified in different clusters to which the pig-derived strains belonged (Figure 3).

## 3. Discussion

This study showed that the proportions of MRSA-positive pigs and farms were approximately 10% and 30%, respectively, in 2022 (Table 1). Different countries exhibit varying prevalence rates of LA-MRSA infections, with European countries, such as Denmark (95% in 2019) and the Netherlands (76% in 2021), exhibiting extremely high rates of confirmed human LA-MRSA infections [14,15]. However, the present study revealed that the LA-MRSA-positive rate in Japan showed an increasing trend over the past five years, highlighting the need to characterize LA-MRSA and to formulate countermeasures against MRSA dissemination to pigs and humans. 

The most common ST type was ST398 (58/88: 65.9%), followed by ST5 (24/88: 27.3%). Other ST8, ST380, ST7096, and new alleles (ST8632) were identified (Table 3). The distribution of STs of MRSA in swine populations varies regionally. In Europe, the United States, and Canada, the primary LA-MRSA strain found in pig production farms was ST398. In addition, swine herds in North America harbored not only ST398, but also a mixed population of LA-MRSA isolates containing ST9 and ST5 [28]. In Asia, except Japan, ST9 isolates dominate [29,30,31]. Although Asai et al. reported in 2012 that methicillin-susceptible *S. aureus* belonging to ST9, a potential reservoir of MRSA, existed in Japan [32], no MRSA belonging to ST9 was detected in the present study. ST5 is the second most common ST type and is a unique characteristic of LA-MRSA in Japan. 

In this study, ST398-*t*034-SCC*mec* Vc (5C2&5) accounted for the largest proportion of isolates, similar to ST398 MRSA isolated from pigs in Europe and north America. The second most common was ST5-*t*002 SCC*mec*IV (2B). ST5-*t*002 is also predominant in the USA, but only a few reports of ST5-*t*002 exist in Europe and other Asian countries [6]. Japan’s importation of parent pigs from Europe and the USA could be a reason for the ST398 and ST5 spread. SNP analysis revealed that MRSA ST398 strains isolated from pigs in Europe and the US were identified in the same cluster as the isolates from pigs in Japan, whereas the ST5 strain isolated from pigs in the USA was not identified in the same cluster as the isolates from Japan. While ST398 has been detected in pigs during animal quarantine, ST5 has not [33]. Therefore, certain ST398 isolates may be of foreign origin, while ST5 isolates may be of unique Japanese origin. However, further analysis is required because the number of analyzed strains is quite limited.

Regarding antimicrobial susceptibility and AMR genes, in addition to all isolates being resistant to MPIPC harboring the *mec*A gene, ST398 showed a high TC resistance rate of 98.3%, harboring tet(K), tet(L), and tet(M), whereas ST5 showed a TC resistance rate of 62.5%, did not possess *tet*(M) genes, and possessed only one of *tet*(K) or *tet*(L). A study indicated that the Tn916 transposon carrying *tet*(M) has been maintained in livestock-associated CC398 since its origin for more than 50 years, largely due to selection pressure from the use of TC in pigs. Regarding isolates from pigs in this study, all *tet*(M) were carried by Tn916 (Supplementary Table S1). 

ST398 possessed approximately 80% of lincosamide/streptogramin (*lsa*(E)/*lnu*(B)/*vga*(A)/*vga*(E)), aminoglycoside (*ant(9)-Ia*/str), and trimethoprim (*dfrG*) resistance genes. Conversely, ST5 had significantly higher macrolide and fluoroquinolone resistance rates than ST398, which harbored the macrolide resistance gene (*erm*(C)), and the mutation gyrA_S84A + parC_S80F mutation responsible for quinolone resistance. ST5 had no *lsa*(E) + *lnu*(B) as a lincosamide/streptogramin resistance gene, but approximately 90% of the isolates had *vag*(A) (Table 4 and Table 5). Thus, ST398 and ST5 exhibited variances in antimicrobial susceptibility and the resistance genes carried. Overall, MRSA possessed resistance genes toward most antimicrobial classes. Antimicrobial use has a strong and positive dose–response relationship with MRSA in pigs and humans living and/or working on pig farms [34]. The continuous promotion of the prudent use of antimicrobials in swine production is required.

ST398 harbored 91.4% of zinc resistance genes (Table 6). Zinc concentration in feed affected LA-MRSA colonization status in pigs. MRSA carriage in pigs is influenced by exposure to therapeutic doses of in-feed ZnO (3000 mg/kg) compared with the recommended dietary levels (100 mg/kg) [35]. In some EU countries, zinc is used for the treatment of swine colibacillosis due to the prudent use of antimicrobial agents, such as colistin. Thus, EU state policies have mandated that zinc usage in therapeutic concentrations should be phased out by 2022 owing to the potential risk associated with zinc supplementation in pig feed for LA-MRSA colonization and spread [36]. Although high zinc concentrations are not approved as veterinary medicinal drugs for prevention or treatment purposes in Japan, restricting zinc utilization to the minimum required as a nutritional ingredient is crucial for preventing MRSA spread. The differences in zinc utilization might contribute to the differences in the LA-MRSA-positive rates observed in pigs between the EU and Japan.

The isolates predominating in Japan, ST398/t034 and SCC*mec*Vc, V, carrying the zinc resistance gene were classified in the same cluster in the SNP analysis; they were isolated from all regions and widely spread throughout the country. Nevertheless, other isolates of the ST, *spa* type, and SCC*mec* types were only endemic in their respective regions. Several factors, such as the system of introduction of pig parents, foster parents, distribution of feed, and transfer of farm workers, may contribute to regional characteristic distribution, but further investigation is needed.

PVL is a leukemolytic toxin comprising two proteins, LukS and LukF. LukS and LukF act by binding to receptors on the leukemic cell membrane surface, forming pores in the membrane and lysing the blood cells [37]. The CC398 strains isolated from humans in Japan possessed both lukF-PV and lukS-PV genes (Figure 1 and Figure 2), but none of the isolates from pigs in Japan had either lukF-PV or lukS-PV. 

Individuals who have contact with pigs, are at higher risk of contracting LA-MRSA from pigs [20,21]. Furthermore, in Europe, MRSA has been detected both in people engaged in pig production and in those who had no contact with livestock. In the Netherlands, LA-MRSA prevalence in humans markedly increased from 2002 to 2007 (from 0% to >20%) [22]. Originally, CC398 was described as colonizing asymptomatic pigs and pig farmers, but several recent studies have reported that CC398 strains are evolving toward increased virulence [38]. The presence of bacteriophage-mediated *scn*, *sak*, and *chp* genes is thought to be a marker of adaptation toward humans, owing to their specificity to the human immune cascade [38,39]. Human- and pig-isolated LA-MRSA isolates acquired phage-encoded immune modulators in Denmark. The present WGS analysis revealed no *scn*, *sak*, *or chp* genes in ST398. LA-MRSA ST398 isolates in Japan have not acquired the ability to cause severe diseases in immunocompetent humans. SNP analysis also showed that MRSA CC398 and MSSA ST398 from humans and MRSA ST398 from pigs formed different clusters, with the MRSA CC398 isolate from humans possessing the immune evasion gene and PVL, but not the zinc resistance gene. Conversely, ST398 from pigs had neither the immune evasion gene nor the PVL gene, but did possess the zinc resistance gene (Figure 1). Overall, these findings indicate that LA-MRSA (ST398) of swine origin is unlikely to spread to humans and cause issues in current clinical practice in Japan. 

Considering pig–human transmission, ST5/t002 MRSA is among the most prevalent lineages causing clinical infections in humans; however, separate and distinct genetic determinants of AMR harbored by clinical ST5 isolates and swine-associated ST5 isolates have been identified in the USA [40]. In the report, the pig-derived isolate was characterized by *erm*(C) and/or *vag*(A) and *tet*(T)/*tet*(L), and the human-derived isolate was characterized by *erm*(A) and had no *tet*(T)/*tet*(L) or vag(A) gene. In this study, ST5 isolates had only *erm*(C) and not *erm*(A), and had *tet*(L) and *vag*(A) genes, similar to the ST5 isolate from swine in the USA and a previous Japanese report [19,40]. Furthermore, our SNP analysis revealed that the Japanese human-origin ST5 strain Mu50 was located in a different cluster from the pig-origin isolate ST5. Therefore, ST5 isolated from pigs in this study could also have a different origin from human strains and could be adapted to pigs.

In this study, ST398 and ST5 were identified as the major genotypes, but ST8, which remained unreported in Japanese pigs, was isolated from pigs. In humans, ST8, particularly the USA300 strain, has been reported to be the most highly virulent and prevalent type and is frequently isolated in the United States [41]. While the CA-MRSA strains reported in Japan are genetically rich in diversity, the Japan-intrinsic CA-MRSA strain (CA-MRSA/J), which is a form of ST8 that differs from USA300, has been isolated since 2003 [42]. A strain of CA-MRSA/J, t1767, carrying SCC*mec* type Ⅳ, leukotoxin gene *lukD* and *lukE*, and genes associated with immune evasion *scn* and *sak* were reported [43,44]. One of the ST8 isolates (R2-S-O9-2-29) from pigs in this study and CA-MRSA/J from humans in Japan had the same characteristics in *spa* type, SCC*me*c type, possession of leukotoxin gene, immune evasion gene, and AMR genes. The SNP analysis revealed a 117-nucleotide difference between the strains R2-S-O9-2-29 and JH4899 (Supplementary Table S2). ST8 was first isolated from pigs in this study in 2020 and is currently a minor population in pigs. Whether this strain was transmitted from humans or other sources to pigs or whether pigs originally harbored it remains unknown. Because a strain with the same genetic characteristics as CA-MRSA, which is pathogenic to humans, was detected in pigs, raising the awareness of workers engaged in pig production becomes crucial. Fundamental hygiene measures such as washing hands, hand sanitizing, changing clothes, and wearing masks in the field engaged in pig production are recommended. Carefully monitoring the potential of ST8 spread in pigs is also crucial. 

## 4. Materials and Methods 

### 4.1. Samples from Pigs at the Slaughterhouse 

In the second half of each fiscal year from 2018 to 2022, sampling was conducted at slaughterhouses located from the northern to the southern part of Japan. The study started with two slaughterhouses in 2018 and was progressively expanded to five slaughterhouses in 2022. Sampling was conducted from pigs after slaughtering, with identification specific to each farm. 

Nasal swabs were collected from 2170 pigs (five per farm) from 434 pig farms. Nasal swabs were obtained from individual slaughtered pigs by inserting a cotton swab (SEEDSWAB No. 1, Eiken Chemical, Tokyo, Japan) into one nostril up to the middle of the swab and rotating it after sanitizing the surface of the nose with alcohol cotton. The collected samples were delivered at 4–8 °C to the laboratory and analyzed within no longer than 5 days after sampling.

### 4.2. Staphylococcus aureus Strain Isolation and MRSA Detection 

Five swabs were taken from each farm and inoculated into 9 mL of Mueller–Hinton broth with 6.5% NaCl, followed by incubation for 16–20 h at 37 °C. Subsequently, 1 mL of pre-enriched broth was transferred into 9 mL of tryptone soy broth (Becton Dickinson, Franklin Lakes, NJ, USA) supplemented with cefoxitin (3.5 mg/L; Sigma-Aldrich, St. Louis, MO, USA) and aztreonam (75 mg/L; Sigma-Aldrich, St. Louis, MO, USA) and incubated for 16–20 h at 37 °C. The broth was then inoculated onto CHROMagarTM MRSA (Kanto Chemical Co., Inc., Tokyo, Japan) or MRSA-selective agar (Becton Dickinson & Co., Franklin Lakes, NJ, USA) and incubated for 24–48 h at 37 °C. Upon the observation of suspected MRSA colonies on these plates, up to five colonies per sample were selected randomly, streaked onto tryptone soy agar plates containing 5% sheep blood (Becton Dickinson), and incubated for 24 h at 37 °C [45]. PCR detections were conducted to identify MRSA by *fem*B gene and *mec*A gene [46]. One of the five isolates was randomly selected for PCR detection to identify MRSA. When the selected isolate was not identified as MRSA, another strain was sequentially tested, and, finally, the isolate identified as MRSA was stored. We confirmed that the stored isolates were *S. aureus* by MALDI-TOF-MS analysis using a MALDI–TOF mass spectrometer (Microflex LT, Bruker Daltonik, Bremen, Germany) with FlexControl software (version 3.4) and MALDI Biotyper (MBT) Compass (version 4.1) for analysis. Among the stored MRSA isolates, one isolate per farm per year was selected for antimicrobial susceptibility testing and whole-genome sequencing (WGS). 

### 4.3. Antimicrobial Susceptibility Testing

Antimicrobial susceptibility testing was performed following the domestic Japanese Veterinary Antimicrobial Resistance Monitoring System. Minimum inhibitory concentrations of antimicrobials were determined for oxacillin, cefoxitin, tetracycline, azithromycin, erythromycin, chloramphenicol, streptomycin, gentamicin, and ciprofloxacin using the broth dilution method following the Clinical and Laboratory Standards Institute (CLSI) guidelines [47]. *S. aureus* ATCC 29213 was used as a quality control strain. The breakpoints of these antimicrobial agents were determined according to CLSI interpretation criteria, except for streptomycin, which was defined microbiologically.

### 4.4. DNA Sequencing for WGS 

DNA samples were prepared for WGS using the DNeasy Blood and Tissue Kit (Qiagen, Valencia, CA, USA). Tagmentation and PCR amplification were performed using a Nextera XT DNA Sample Preparation Kit (Illumina, Inc., San Diego, CA, USA). WGS was carried out using a MiSeq instrument (Illumina, Inc., San Diego, CA, USA) in paired-end mode (2 × 300). Quality control and trimming of MiSeq raw reads were performed with fastp (v0.23.2) (Chen et al., 2018). De novo assembly was performed using Shovill software (version 1.1.0) (https://github.com/tseemann/shovill (accessed on 10 February 2023)). Quality assessment for genome assemblies was evaluated using QUAST (https://doi.org/10.1093/bioinformatics/btt086 (accessed on 27 June 2023)).

The contig sequences were analyzed using AMR FinderPlus (version 3.11.2) (https://github.com/ncbi/amr (accessed on 10 February 2023)) and abricate (version 1.0.1) (https://github.com/tseemann/abricate (accessed on 10 February 2023)) to infer the antimicrobial resistance (AMR) genes, Panton–Valentine leukocidin (PVL) toxin gene, and bacteriophage-mediated immune evasion genes (*sak*, *chp*, and *scn* genes). 

The zinc-resistant gene *czrC* was determined using Prokka (version 1.14.6) (https://github.com/tseemann/prokka (accessed on 9 May 2023)) and GenAPI (version 1.0) (https://github.com/MigleSur/GenAPI (accessed on 9 May 2023)). WGS data were also used for MLST, *spa* typing, and SCC*mec* typing. MLST analysis was performed using FastMLST (version 0.0.15) (https://github.com/EnzoAndree/FastMLST (accessed on 9 May 2023)). *Spa* typing was performed using the Python script (get_spa_type.py) (version 0.1.0) (https://github.com/mjsull/spa_typing (accessed on 9 May 2023)), and SCC*mec* typing was performed using SCCmecFinder (version 1.2) (https://cge.food.dtu.dk/services/SCCmecFinder/ (accessed on 7 August 2023)). Snippy (version 4.6.0) (https://github.com/tseemann/snippy (accessed on 9 May 2023)) was used to generate a core genome alignment based on single-nucleotide polymorphisms (SNPs) and insertion/deletion (indels). The chromosome of the MRSA strain isolated from a human patient in Japan, N1195 (CC398 (ST1232)) and Mu50 (ST5), was used as a reference genome. The chromosomes of the methicillin-susceptible *S. aureus* strain (ST398) isolated from human patients in Japan (SA0052, SA0479, and SA2854) and the MRSA strain isolated from pigs in Europe (55-100-120 and 55-103-051) and USA (ISU933 and P23-03_SW181_1) were used (Supplementary Table S1). Gubbins (version 2.3.4) (https://github.com/nickjcroucher/gubbins (accessed on 9 May 2023)) was used to remove recombination from the resulting alignment (Croucher et al., 2014). The phylogenetic tree was finally visualized using ggtree (version 3.8.0) (https://bioconductor.org/packages/release/bioc/html/ggtree.html (accessed on 18 June 2023)). 

### 4.5. Statistical Analyses 

Fisher’s exact test was used to identify differences in the MRSA-positive farm and sample rates between 2018 and the other year and the resistance rates between ST398 and ST5. A probability of *p* < 0.05 was considered statistically significant [48]. 

## 5. Conclusions

Positive LA-MRSA rates on the farms and samples in Japan remained lower than those observed in European countries, where LA-MRSA had been dismissed in human clinical sites, but the rate showed a gradually increasing trend over five years. ST398/t034 was predominant, followed by ST5/t002. ST398 and ST5 demonstrated differences in antimicrobial susceptibility and the resistance genes carried. Overall, LA-MRSA possessed genes resistant to many antimicrobial classes. Moreover, ST398 harbored 91.4% zinc resistance genes. These findings indicate that the co-selection pressure associated with multidrug and zinc resistance may have markedly contributed to LA-MRSA persistence. Therefore, the effective prevention and control of LA-MRSA dissemination requires utilizing antimicrobials prudently and limiting zinc usage to the minimum required as a nutrient. Furthermore, the immune evasion genes, *scn*, *sak*, and *chp*, were undetected in ST398 and ST5. The SNP analysis revealed that the CC398 from humans and ST398 from pigs, as well as ST5 from humans and pigs, formed different clusters. ST8 is a minor genotype among pig-derived strains, but a major genotype in CA-MRSA in Japan. An isolate in this study was identical in genotype to a strain isolated from a clinical patient in Japan, despite more than 100 nucleotide differences being identified in the SNP analysis. Consequently, further monitoring is essential to prevent further transmission from humans and other sources to pigs, the spread of ST8 among pigs, and the subsequent negative impact on human health. Although LA-MRSA of swine origin is currently unlikely to spread to humans and become a problem in clinical practice, the effective prevention of LA-MRSA transmission from pigs to humans and from humans, pigs, and other sources to pigs in Japan requires the implementation of consistent fundamental hygiene measures in the field engaged in pig production.

## Figures and Tables

**Table 1:** Results of MRSA detection in all slaughterhouses studied.

	2018 *	2019 *	2020 *	2021 *	2022 *
Number of slaughterhouses	2	3	4	5	6
Number of pigs sampled	240	375	465	515	575
Number of positive samples	7	24	29	39	57
Positive rate of samples	2.9%	6.4%	6.2%	7.6% **	9.9% **
Number of farms	48	75	93	103	115
Number of positive farms	4	10	19	21	34
Positive rate of farms	8.3%	13.3%	20.4%	20.4%	29.6% **

* Fiscal year (spanning from April to March of the subsequent year), ** *p*-values: positive rate vs. 2018 determined by Fisher’s exact test. ** *p* < 0.05.

**Table 2:** Results of MRSA detection in 2 slaughterhouses continuously surveyed for 5 years among the five slaughterhouses listed in Table 1.

	2018 *	2019 *	2020 *	2021 *	2022 *
Number of slaughterhouses	2	2	2	2	2
Number of pigs sampled	240	300	340	340	320
Number of positive samples	7	20	20	24	23
Positive rate of samples	2.9%	6.7% **	5.9%	7.1% **	7.2%**
Number of farms	48	60	68	68	64
Number of positive farms	4	7	13	12	15
Positive rate of farms	8.3%	11.7%	19.1%	17.6%	23.4% **

* Fiscal year (spanning from April to March of the subsequent year), ** *p*-values: positive rate vs. 2018 determined by Fisher’s exact test. ** *p* < 0.05.

**Table 3:** ST type, *spa* typing, and SCC*mec* of MRSA isolated from pig.

ST	*Spa* Typing			SCC*mec*		
		Number	Rate (%)		Number	Rate (%)
ST398	t034	48	54.5	Vc (5C2&5)	40	45.5
	t571	6	6.8	V (5C2&5)	4	4.5
	t21390	1	1.1	V (5C2)	2	2.3
	UD	3	3.4	Va (5C2)	2	2.3
	t21388			I (1B) or IV (2B)	5	
				Untypable	5	
		58	65.9		58	65.9
ST5	t002	10	11.4	IV (2B)	12	13.6
	t21388	10	11.4	IV (2B&5)	6	6.8
	t010	2	2.3	V (5C2&5)	3	3.4
	t1560	2	2.3	Untypable	3	3.4
		24	27.3		24	27.3
ST8	t21389	1	1.1	IV(2B)	3	3.4
	t1767	1	1.1			0.0
	t5071	1	1.1			0.0
		3	3.4		3	3.4
ST380	t024	1	1.1	IVc (2B)	1	1.1
		1	1.1		1	1.1
ST7096	t9624	1	1.1	IVc (2B)	1	1.1
		1	1.1		1	1.1
ST8632	t034	1	1.1	Untypable	1	1.1
		1	1.1		1	1.1
	total	88			88	

**Table 4:** Antimicrobial resistance prevalence of MRSA isolated from pig.

		Resistant Rate (%)			
Antimicrobial Agents	BP	Total (n = 88)	ST398 (n = 58)	ST5 (n = 24)	*p*-Values Determined by Fisher’s Exact Test (ST398 vs. ST5)
MPIPC	4	100	100	100	-
CFX	8	100	100	100	-
TC	16	85.2	98.3	62.5	*p* < 0.05
AZM	8	46.6	37.9	66.7	*p* < 0.05
EM	8	46.6	37.9	66.7	*p* < 0.05
CP	32	43.2	41.4	54.2	-
SM	64	31.8	36.2	20.8	-
GM	16	2.3	0	0	-
CPFX	4	18.2	8.6	33.3	*p* < 0.05

*p*-values were determined by Fisher’s exact test. *p* < 0.05.

**Table 5:** AMR genes identified in MRSA isolated from pig.

	Antimicrobial Resistance Genes/	Total (n = 88)		ST398 (n = 58)		ST5 (n = 24)	
Antimicrobial Class	Mutation	Number	Rate (%)	Number	Rate (%) in ST398	Number	Rate (%) in ST5
beta-lactam	*mecA*	88	100	58	100	24	100
tetracycline	*tet*(K)	7	8.0			6	25.0
	*tet*(L)	8	9.1			8	33.3
	*tet*(M)	11	12.5	10	17.2		
	*tet*(K) + *tet*(M)	47	53.4	46	79.3		
	*tet*(L) + *tet*(M)	2	2.3	2	3.4		
		75	85.23	58	100	14	58.3
macrolide	*erm*(A)	4	4.5	3	5.2		
	*erm*(C)	40	45.5	22	37.9	17	70.8
		44	50.0	25	43.1	17	70.8
lincosamide/	*vga*(A)	24	27.3	2	3.4	22	91.7
streptogramin	*vga*(E)	2	2.3	2	3.4		
	*lsa*(E) + *lnu*(B)	36	40.9	36	62.1		
	*lsa*(E) + *lnu*(B) + *vga*(A)	1	1.1	1	1.7		
	*lsa*(E) + *lnu*(B) + *vga*(E)	5	5.7	5	8.6		
	*lsa*(E) + *vga*(A)	2	2.3		0.0	2	8.3
		70.0	79.5	46.0	79.3	24	100.0
phenicol	*catA*	3	3.4	3	5.2		
	*fexA*	32	36.4	20	34.5	12	50.0
		35	39.8	23	39.7	12	50.0
aminoglycoside	*aadD1*	6	6.8			6	25.0
	*aadD1* + *ant(6)-Ia* + *spw*	1	1.1			1	4.2
	*aadD1* + *ant(6)-Ia* + *spw* + *str*	1	1.1			1	4.2
	*ant(9)-Ia*	31	35.2	30	51.7		
	*ant(9)-Ia* + *str*	15	17.0	15	25.9		
	*aac(6′)-Ie/aph(2″)-Ia*	2	2.3				
	*str*	4	4.5	3	5.2	1	4.2
		60	68.2	48.0	82.8	9	37.5
trimethoprim	*dfrG*	49	55.7	46	79.3	3	12.5
		49	55.7	46	79.3	3	12.5
quinolone	*gyr*A_S84A + *par*C_S80F	7	8.0			7	29.2
	*gyrA*_S84L + *parC*_S80F	5	5.7			2	8.3
	*gyrA*_S84L + *parC*_S80Y	4	4.5	4	6.9		
	*parC*_S80F	7	8.0	3	5.2	4	16.7
		23	26.1	7	12.1	13	54.2

**Table 6:** Zinc chloride resistance genes and leucocidin toxin genes detected in MRSA isolated from pig.

		Total		ST398		ST5	
		Number	Rate (%)	Number	Rate (%)	Number	Rate (%)
*czrC*	+	56	63.6	53	91.4	2	8.3
	−	32	36.4	5	8.6	22	91.7
*LukD + LukE*	+	29	33.0	0	0	24	100
	−	59	67.0	58	100		0

## Data Availability

All raw short-read sequence data have been deposited in the DNA Data Bank of Japan (BioSample IDs, SAMD00326077 to SAMD00326107 and SAMD00407345 to SAMD00407369; DRA accession no. DRA012277 and DRA012899, Supplementary Table S1).

## Supplementary Materials

The following supporting information can be downloaded at: https://www.mdpi.com/article/10.3390/antibiotics13020155/s1: Table S1: Accession number and genetic profiles of methicillin-resistant *Staphylococcus aureus* (MRSA) analyzed in this study; Table S2: Single-nucleotide polymorphism (SNP) distance matrix of ST8 strains.
